# Barriers and Enablers of Cervical Cancer Screening Among Saudi Women

**DOI:** 10.7759/cureus.67720

**Published:** 2024-08-25

**Authors:** Hala Aljohani, Amani Alsaedi

**Affiliations:** 1 Preventive Medicine, Ministry of Health, Jeddah, SAU; 2 Preventive Medicine, Ministry of Health, Makkah, SAU

**Keywords:** enablers, barriers, saudi women, screening uptake, cervical cancer

## Abstract

Background: Cervical cancer is a significant global health issue, with low screening uptake rates in many regions, including Saudi Arabia. This study sought to understand the barriers and enablers of cervical cancer screening among Saudi women, particularly in Makkah, Saudi Arabia.

Objectives: To identify barriers and enablers influencing cervical cancer screening uptake among Saudi women.

Methods: This cross-sectional study was conducted in Makkah, Saudi Arabia, among 418 Saudi women aged 21-65. A questionnaire collected data on demographics, reproductive health, service utilization, and attitudes toward cervical cancer screening. Statistical tests such as the chi-square test and Mann-Whitney test were used for inferential analyses to determine significance, with a p-value of less than 0.05 considered significant.

Results: The study identified significant factors influencing cervical cancer screening uptake, including age, marital status, and household monthly income (p<0.001, p<0.001, and p=0.018, respectively). Health conditions such as hypertension, dyslipidemia, and miscarriage history also showed significant associations with screening uptake (p<0.001, p=0.009, and p<0.001, respectively). Additionally, the availability of screening services and encouragement from healthcare providers were linked to higher screening rates (p<0.001 and p=0.006, respectively). However, perceived benefits, barriers, susceptibility, and seriousness were not significantly associated with screening uptake (p>0.05).

Conclusions: The study identified several demographic and healthcare service utilization factors influencing cervical cancer screening uptake among Saudi women. These findings can be instrumental in informing public health measures, awareness campaigns, and healthcare policies aimed at increasing cervical cancer screening rates in Saudi Arabia.

## Introduction

Cervical cancer is a significant health concern globally, being the fourth most common cancer in women, accounting for 660,000 new cases and 350,000 deaths in 2022 [1]. Studies have highlighted disparities in cervical cancer incidence and mortality, particularly affecting racial/ethnic minorities, sexual/gender minorities, individuals with disabilities, and those from low-income or geographically defined populations [2]. WHO report shows the majority of cervical cancer-related mortality (90%) occurred in low- and middle-income countries [1]. As of 2022 in Saudi Arabia, the crude incidence of cervical cancer per 100,000 women was 2.4 and the mortality rate was 1.3 [3]. There is a projected increase in the incidence of cervical cancer in the country, with an estimated 309 new cases and 117 deaths anticipated by 2025 [4].

Research has identified various risk factors for developing cervical cancer, including early onset of sexual activity, smoking, having multiple sexual partners, long-term use of hormonal contraceptives, high parity, and a history of sexually transmitted infections [5,6]. Human papillomavirus (HPV) testing and regular screening for women aged 21- 65 years have been suggested to reduce the incidence and mortality of cervical cancer [2]. Despite the efforts made to increase awareness and screening for cervical cancer, 40% of women diagnosed with cervical cancer in Saudi Arabia are identified at an advanced stage, emphasizing the necessity for improved early detection strategies [5]. Research indicates that only a small proportion of Saudi females have good knowledge about cervical cancer, highlighting a gap in awareness that may affect prevention and early intervention efforts [7].

The incidence of cervical cancer in Saudi Arabia has been reported by some studies to be 2.1 per 100,000 women [8]. Bondagji et al. (2013) have shown a high prevalence of high-risk HPV infections in Saudi women attending gynecologic clinics with HPV genotypes 16 and 18 being the most common in women with cervical cancer [9]. Despite the efforts to raise awareness, only a small proportion of Saudi women have good knowledge about cervical cancer [7].

Factors such as increasing age, education level, income, perceived risk of cervical cancer, source of information, having a family doctor, and previous gynecological history are associated with higher screening rates [10,11], whereas negative beliefs related to screening, modesty, financial issues, and embarrassment in discussing gynecological or sexual diseases have been identified as barriers to cervical cancer screening programs among Arab Muslim women, indicating cultural and personal inhibitions as key obstacles [5,12]. Additionally, the limited understanding of the Pap smear test, cervical cancer risk factors, implications, and prevention methods among women in Saudi Arabia underscores a critical gap in knowledge that may hinder screening practices [13,14].

Various validated questionnaires have been used to explore barriers and enablers of HPV vaccine uptake and cervical cancer screening among Saudi women. Hussain et al. in 2016 assessed awareness of HPV infection and vaccine acceptance among young Saudi women [15]. Alshammiri in 2022 evaluated 387 female participants' knowledge of HPV and its vaccine using a 26-item questionnaire [16]. Rezq et al. in 2023 conducted a cross-sectional study on women's knowledge and acceptance of HPV vaccination and cervical cancer screening [17]. Al-Amro et al. in 2020 and Alshehri et al. in 2024 used a validated three-part questionnaire to study cervical cancer screening factors among family medicine physicians and community women in Riyadh [14,18].

The findings on cervical cancer screening practices among women in Saudi Arabia highlight several barriers and enablers to screening uptake. One significant barrier is the lack of awareness and knowledge about HPV infection and its association with cervical cancer among both healthcare professionals and the general population [10,12,16,19,20]. Rezq et al. (2023) showed that 78.9% of the participants had never heard of cervical cancer, 65.6% had never heard of cervical cancer screening, only 31.1% had heard of the HPV vaccine, and only 8.3% of participants were screened for cervical cancer [17]. According to a study by Ghazi et al. (2023), only 10.2% of family doctors recommended Pap smear tests to their female patients [11]. Another study by Alshehri et al. (2024) shows that the rate of Pap smear test uptake among family medicine physicians was only 20.6%, compared to 30.7% among women in the community. Although this finding did not achieve statistical significance (p=0.067), it highlights the attitude of Saudi women, even educated ones, toward cervical cancer screening [14].

This study addresses the need to understand the factors affecting cervical cancer screening uptake among women in Makkah, Saudi Arabia, in 2024. It explores sociocultural influences, perceptions impact, and healthcare access. The findings will inform measures to increase screening rates and reduce the incidence of this preventable disease.

## Materials and methods

Study design and population

This analytical cross-sectional study was conducted in Makkah, Saudi Arabia, in 2024. The study's inclusion criteria were Saudi females residing in Makkah, aged between 21 and 65 years old, and showing willingness to participate in the study. This specific age group was chosen in alignment with the cervical cancer screening recommendations in the Kingdom of Saudi Arabia. Excluded participants were non-Saudi women, those living outside Makkah, and women outside the specified age range.

Convenience sampling was used for this study, an approach suitable given the specific demographic and location requirements of the target population. This non-probability sampling technique was chosen due to its feasibility and the accessibility of the participants. The estimated sample size for this study was 385, as calculated using an online sample size calculator (Raosoft.com). This figure takes into account a 5% margin of error, a 95% confidence level, and a 50% response distribution. A total of 418 participants were recruited.

Data collection technique and tool

Data collection for this study utilized a structured questionnaire to identify enablers and barriers of cervical cancer. The questionnaire was distributed through a web-based platform. This questionnaire was carefully chosen due to its proven reliability and validity. Its effectiveness had been demonstrated in previous studies conducted in Riyadh, Saudi Arabia, and Jordan, which had similar settings to this study [14,18]. Participants who met the criteria for inclusion were invited, and their consent was obtained before collecting any data.

The questionnaire was divided into several sections. The first section collected sociodemographic and reproductive data information. The second section focused on health service utilization. The third section assessed participants’ knowledge and attitudes toward cervical cancer screening through the perceived benefits, barriers, susceptibility, and seriousness scales. This section utilized a four-point Likert scale, where 4 signified 'strongly agree' and 1 signified 'strongly disagree', to evaluate women's perception of cervical cancer and its screening. This section included a five-item scale for perceived benefits (scores ranging from 5 to 20), a 10-item scale for perceived barriers (scores ranging from 10 to 40), a four-item scale for perceived susceptibility to cervical cancer (scores ranging from 4 to 16), and a six-item scale for perceived seriousness regarding the severity of cervical cancer (scores ranged from 6 to 24).

Statistical analysis

The data was analyzed using IBM SPSS Statistics (version 29.0). Categorical variables were represented as proportions. For inferential analyses, statistical tests such as the chi-square test and Mann-Whitney test were used. Significance was established at p-values less than 0.05, and conclusions were made with a 95% confidence level.

Ethical considerations

Before data collection, ethical approval (IRB number: 0708-210424) was secured from the Institutional Review Board (IRB) at the Security Forces Hospital Program in Holy Capital. Participants were given a clear description of the study's nature and objective to obtain their consent prior to filling out the questionnaire. All data was treated with confidentiality, stored securely, and used strictly for research purposes.

## Results

A total of 418 respondents met the inclusion criteria and were included in the analysis. Table 1 elucidates the demographic information of the 418 participants in the study. A large proportion of the population were 30 years or younger (35.2%), providing a young demographic for the study. Most were married (56.2%), and 49.3% of the participants had a household monthly income of 10,000 Saudi Riyals or less, suggesting a moderate economic status among the participants. It is also noteworthy that a majority of the participants (56.5%) were employed at the time of the study.

**Table 1:** Demographic characteristics Demographic characteristics of the study participants including miscarriage history and family history of cervical cancer are presented in the table.

n=418		N	%
Age (years)	≤30	147	35.2%
	31-40	105	25.1%
	41-50	79	18.9%
	>50	87	20.8%
Marital status	Single	121	28.9%
	Married	235	56.2%
	Divorced	37	8.9%
	Widowed	25	6.0%
Household monthly income (Saudi Riyals)	≤10,000	206	49.3%
	10,001-20,000	116	27.8%
	>20,000	96	23.0%
Currently employed		236	56.5%
Diabetes mellitus		28	6.7%
Hypertension		48	11.5%
Dyslipidemia		61	14.6%
Miscarriage history	No miscarriage	228	54.5%
	Less than three miscarriages	172	41.1%
	Three or more miscarriages	18	4.3%
Family history of cervical cancer	Yes	60	14.4%
	No	358	85.6%

The comorbidities and family history of the participants were also reported. Out of the 418 participants, only 6.7% had diabetes mellitus, 11.5% had hypertension, and 14.6% had dyslipidemia. Additionally, 54.5% of the participants reported no history of miscarriage, while 41.1% reported having less than three, and 4.3% reported having three or more miscarriages. Regarding the family history of cervical cancer, only 14.4% of the participants reported a positive family history, while the majority (85.6%) reported no family history of cervical cancer (Table 1).

Table 2 provides insights into healthcare access and service utilization among participants. A significant proportion of participants (57.2%) did not possess health insurance, indicating potential barriers to healthcare access. Only 39.7% of participants reported that cervical cancer screening services were available in their health sector, highlighting a potential gap in healthcare provision. Interestingly, about half of the participants (51.2%) reported that healthcare providers encouraged them for cervical cancer screening uptake, demonstrating a proactive approach from healthcare providers. According to the data, out of the 418 participants, 195 (46.7%) reported that they have had a cervical cancer screening (Pap smear), while 223 (53.3%) reported that they have not.

**Table 2:** Healthcare access and service utilization This table shows healthcare access and service use, including health insurance, cervical cancer screening availability, provider encouragement, impact of male physicians on screening, and past Pap smears.

n=418		N	%
Health insurance	Yes	179	42.8%
	No	239	57.2%
Is there cervical cancer screening services (Pap test) in your health sector?	Yes	166	39.7%
	No	149	35.6%
	Never asked	103	24.6%
Did healthcare providers encourage you for cervical cancer screening uptake (Pap test)	Yes	214	51.2%
	No	204	48.8%
Presence of male physician prevents you from Pap test uptake	Yes	200	47.8%
	No	218	52.2%
Have you ever had a cervical cancer screening (Pap smear)?	Yes	195	46.7%
	No	223	53.3%

Table 3 presents the associations between demographic factors and cervical cancer screening uptake, revealing several significant associations. Age, marital status, and household monthly income were all significantly associated with cervical cancer screening uptake (p<0.001, p<0.001, and p=0.018, respectively). Additionally, the presence of hypertension, dyslipidemia, and miscarriage history were also significantly associated with cervical cancer screening uptake (p<0.001, p=0.009, and p<0.001, respectively).

**Table 3:** Demographic factors associated with Pap smear uptake Chi-Square test. *Association is significant at p-value <0.05.

n=418		Have you ever had a cervical cancer screening (Pap smear)?								
		Yes			No					
		N	%		N		%			p-value
Age (years)	≤30	41		21.0%		106		47.5%	<0.001*	
	31-40	41		21.0%		64		28.7%		
	41-50	45		23.1%		34		15.2%		
	>50	68		34.9%		19		8.5%		
Marital status	Single	35		17.9%		86		38.6%	<0.001*	
	Married	124		63.6%		111		49.8%		
	Divorced	21		10.8%		16		7.2%		
	Widowed	15		7.7%		10		4.5%		
Household monthly income (Saudi Riyals)	≤10,000	82		42.1%		124		55.6%	0.018*	
	10,001-20,000	64		32.8%		52		23.3%		
	>20,000	49		25.1%		47		21.1%		
Employment status	Yes	110		56.4%		126		56.5%	1.00	
	No	85		43.6%		97		43.5%		
Diabetes mellitus	Yes	16		8.2%		12		5.4%	0.327	
	No	179		91.8%		211		94.6%		
Hypertension	Yes	36		18.5%		12		5.4%	<0.001*	
	No	159		81.5%		211		94.6%		
Dyslipidemia	Yes	38		19.5%		23		10.3%	0.009*	
	No	157		80.5%		200		89.7%		
Miscarriage history	No miscarriage	83		42.6%		145		65.0%	<0.001*	
	Less than three miscarriages	100		51.3%		72		32.3%		
	More than three miscarriages	12		6.2%		6		2.7%		
Family history of cervical cancer	Yes	27		13.8%		33		14.8%	0.889	
	No	168		86.2%		190		85.2%		

Table 4 further explores the associations between healthcare access and service utilization factors and cervical cancer screening uptake. The availability of cervical cancer screening services in the health sector (p<0.001), encouragement from healthcare providers for cervical cancer screening uptake (p=0.006), and the presence of a male physician (p=0.024) all had significant associations with cervical cancer screening uptake.

**Table 4:** Healthcare access and service utilization factors associated with Pap smear uptake Chi-Square test. *.Association is significant at p-value <0.05.

n=418		Have you ever had a cervical cancer screening (Pap smear)?					
		Yes		No			
		N	%	N	%	p-value	
Health insurance	Yes	88	45.1%	91	40.8%	0.428	
	No	107	54.9%	132	59.2%		
Is there cervical cancer screening services (Pap test) in your health sector?	Yes	103	52.8%	63	28.3%	<0.001*	
	No	71	36.4%	78	35.0%		
	Never asked	21	10.8%	82	36.8%		
Did healthcare providers encourage you for cervical cancer screening uptake (Pap test)	Yes	114	58.5%	100	44.8%	0.006*	
	No	81	41.5%	123	55.2%		
Presence of male physician prevents you from Pap test uptake	Yes	105	53.8%	95	42.6%	0.024*	
	No	90	46.2%	128	57.4%		

Table 5 investigates the association between cervical cancer screening uptake and perceived benefits, barriers, susceptibility, and seriousness. Despite measurements across these four factors, no significant associations were found between these and cervical cancer screening uptake.

**Table 5:** Association between Pap smear uptake and perceived benefits, barriers, susceptibility, and seriousness Mann-Whitney test.

	Have you ever had a cervical cancer screening (Pap smear)?	N	Mean rank	Sum of ranks
Perceived benefits	Yes	195	208.22	40603.00
	No	223	210.62	46968.00
	p-value = 0.838	418		
Perceived barriers	Yes	195	200.00	38999.50
	No	223	217.81	48571.50
	p-value = 0.131	418		
Perceived susceptibility	Yes	195	208.15	40589.00
	No	223	210.68	46982.00
	p-value = 0.827	418		
Perceived seriousness	Yes	195	215.57	42035.50
	No	223	204.20	45535.50
	p-value = 0.331	418		

## Discussion

This study examined the enablers and barriers of cervical cancer screening among 418 participants in Makkah, Saudi Arabia. It analyzes various demographic characteristics, healthcare access, and service utilization factors. The results showed that 46.7% of women in the study had a Pap smear test. This finding shows an increase in Pap smear uptake among Saudi citizens compared with previous national studies. A recent cross-sectional study by Ghazi et al. (2023) showed that 33.5% of 665 women studied in Jeddah had a Pap smear taken [11]. Another cross-sectional study conducted in Riyadh in 2018 found that among 450 women studied, 26% had ever undergone a Pap smear [5]. A study conducted in Al Hassa in 2017 showed that only 17.2% of 506 participants had a Pap smear taken [21]. Although there has been a steady increase in Pap smear uptake, there is still a need to understand the root causes of this underutilization of screening services and develop targeted interventions to increase awareness and uptake.

The majority of participants in this study were young (35.2% ≤30 years), married (56.2%), had a monthly household income of ≤10,000 Saudi Riyals (49.3%), and were employed (56.5%). This is similar to findings in a previous study by Ghazi et al. (2023), which showed that 4.4% of their study participants were single, and 48.6% earned under 4,000 Saudi Riyals [11]. A similar finding was also reported by Bayu et al. (2016) who found that Ethiopian women in the age range of 30-39 years were about 1.789 times more likely to be screened for cervical cancer compared with those 21-29 years old [22]. Destaw et al. (2021) found a similar age range of Ethiopian women who sought cervical cancer screening [23]. Furthermore, our study showed that Pap smear screening was higher among older females (>40 years) than among younger females, which is corroborated by a study in Kenya that reported low screening rates among younger Kenyan women [24].

A study by Mukama et al. (2017), in contrast to our study, found that having a higher monthly income was associated with better knowledge and attitudes toward cervical cancer prevention, indicating that socioeconomic factors play a role in screening behaviors [25]. Elgzar et al. (2022) found similar findings in a cross-sectional study among Saudi women [26]. Another demographic factor related to income but not explicitly measured in our study is the level of education. Alnafisah et al. (2019), in a study among Saudi women, reported that most participants with moderate knowledge about cervical cancer were highly educated, suggesting a positive correlation between education level and knowledge about screening [6].

The comorbidities associated with increased Pap smear screening in this study included hypertension and dyslipidemia. The reason for the increased rate of Pap smear screening among these groups could be regular doctor visits which might increase the chance of doctor recommendations [10,11]. A study by Ackerson and Gretebeck (2007) highlighted that women without a usual source of healthcare were less likely to obtain regular cervical cancer screening, emphasizing the importance of access to healthcare services [27]. Other comorbidities associated with increased Pap smear uptake in our findings included a history of miscarriage; a family history of cervical cancer was not found to have a statistically significant association in our study. These findings are consistent with those of Alsalmi and Othman (2022) who mentioned that previous gynecologic examinations and history of abortions were highly associated with Pap smear uptake [10].

This study also highlights the significance of healthcare providers in influencing screening behaviors. Out of the 214 women who were encouraged and informed by a physician to do a Pap smear, 114 ended up having the screening; meanwhile, out of the 204 women in our study who were not encouraged by their physician, only 81 ended up having the screening done. Information regarding Pap tests and regular visits to family physicians were found to be influential factors in previous studies [10,11]. This emphasizes the importance of healthcare provider counseling and encouragement in promoting screening uptake.

Despite the proactive role of healthcare providers, our study revealed several barriers to cervical cancer screening, including the presence of male physicians, insufficient screening services in the local health sector, and younger age. In Saudi Arabia, cultural norms often favor female healthcare providers for intimate procedures, making male physicians a barrier due to discomfort or reluctance among women. Limited availability of screening services further restricts access, while younger women may lack awareness or feel less urgency for preventive measures. Findings on male physicians performing cervical cancer screening have varied results; while a study by Kaneko (2018) found Japanese women to have confidence in the results given by a male physician, Marques et al. (2023) reported that one of the barriers for women to seek a Pap smear test was having to face a male physician [28,29]. These findings emphasize the significance of increasing awareness of cervical cancer screening and making efforts to enhance accessibility and streamline screening services. Previous studies have mentioned that a significant portion of women did not perceive a need for a Pap test; however, we did not find any statistically significant difference in perceived benefits, risks, barriers, or seriousness among individuals who had Pap smear screening and those who did not.

Cervical cancer screening guidelines aim to detect precancerous lesions early to prevent the development of cervical cancer. National guidelines, such as those developed in Saudi Arabia, emphasize the importance of routine screening and treatment of precancerous lesions [4]. In countries like Iran, improving access to cervical cancer screening and treatment services is crucial. Priority setting involves making these services accessible and affordable for all women, along with establishing population-based cancer registries and cervical screening registries for effective monitoring and evaluation of screening programs [30]. For Muslim societies, recommendations for cervical cancer screening often draw from successful international models. Countries like Australia, Canada, Europe, the Netherlands, New Zealand, Singapore, the United Kingdom, and the USA have been cited for their organized screening programs that have shown positive outcomes in controlling cervical cancer incidence [31].

Recommendations

Based on the study findings, we recommend the development of targeted interventions to increase cervical cancer screening uptake, especially among younger females. These could include educational campaigns focusing on the importance of early screening and addressing misconceptions about the process. Furthermore, healthcare providers should be encouraged to promote cervical cancer screening during their interactions with patients. The availability of female physicians for screening should be increased to address the discomfort some women feel with male physicians. Finally, efforts should be made to improve the accessibility of screening services in all health sectors.

Strengths

One of the main strengths of this study is its comprehensive investigation of various factors influencing cervical cancer screening uptake. This includes demographic characteristics, healthcare access, and service utilization, providing a holistic understanding of the issue. The use of a structured questionnaire with proven reliability and validity also enhances the strength of the study. Additionally, the relatively large sample size of 418 participants adds to the robustness of the research.

Limitations

Despite its strengths, the study also has some limitations. Its cross-sectional design means that causality cannot be established. Furthermore, the use of convenience sampling may limit the generalizability of the findings. As the study was conducted in Makkah, Saudi Arabia, the results may not be representative of other regions in the country or in other countries. Finally, the study relied on self-reported data, which may be subject to reporting bias.

## Conclusions

This study offers valuable insights into the factors that enable and hinder cervical cancer screening among women in Makkah, Saudi Arabia. It reveals that 46.7% of participants have undergone a Pap smear test, suggesting a rising trend in screening uptake compared to past studies. Key demographic attributes like age, marital status, and household income significantly affect screening behaviors. The study also underlines the crucial role of healthcare providers in encouraging screening. However, barriers like the presence of male physicians and inadequate screening services still exist. Consequently, the study emphasizes the need for focused efforts to increase cervical cancer screening, especially among younger women, and to enhance the availability of screening services.
